# Validation of the Chinese version of the Oslo-3 Social Support Scale among nursing students: a study based on Classical Test Theory and Item Response Theory models

**DOI:** 10.1186/s12912-024-02033-5

**Published:** 2024-05-30

**Authors:** Dongmei Zhang, Ting Yuan, Anle Huang, Xiaoping Li, Liu Yang, Congzhi Wang, Mingming Liu, Yunxiao Lei, Lu Sun, Jing Li, Lin Zhang, Jing Zhang

**Affiliations:** 1 School of Nursing, Wannan Medical College, 22 Wenchang West Road, Yijiang District, Wuhu City, Anhui Province P.R. China; 2 Nursing Department, the People’s Hospital of Yingshang, 566 Ganluo Road, Chengbei New District, Yingshang County, Anhui Province P.R. China

**Keywords:** Social support, Reliability, Validity, Discrimination, Difficulty

## Abstract

**Background:**

Nursing students are encountering a range of health issues. Assessing social support is a key component in most questionnaire surveys related to health status, aiming to investigate the relationships and mechanisms between health status and social support to enhance overall health. Therefore, it is essential to seek out appropriate instruments to evaluate social support for nursing students. The Oslo-3 Social Support Scale (OSSS-3) is a reliable and concise instrument for evaluating social support. To date, there have been no studies validating the OSSS-3 based on Item Response Theory (IRT) models. Also, an officially validated Chinese version has not been found. The current research intended to verify the Chinese version of the OSSS-3.

**Methods:**

The OSSS-3 was translated into Chinese and culturally adapted. Subsequently, the OSSS-3 was validated by employing the Classical Test Theory (CTT) and IRT models.

**Results:**

The split-half reliability was 0.622. The Cronbach’s α coefficient was 0.687. The correlations between each item and total scores varied from 0.723 to 0.835. The retest coefficient was 0.907. The content validity index was 0.933. A single common factor was extracted and accounted for 61.559% of the variance. The item loading values on the single factor were between 0.743 and 0.814. The communalities were between 0.552 and 0.663. There was no variance between males and females (*P* = 0.055). The difference in scores between the top (30%) and bottom (30%) groups attained significance. IRT models results revealed that the discrimination parameters ranged from 1.39 to 2.33 and difficulty parameters increased monotonically.

**Conclusion:**

The OSSS-3 demonstrates satisfying psychometric properties and is a proper instrument for measuring social support in Chinese nursing students.

## Introduction

Social support includes perceived and received support [1]. The former refers to an individual’s subjective evaluation of the availability of assistance when required, whereas the latter pertains to actual assistance provided to those in need [1, 2]. Social support is multidimensional, encompassing two sub-categories (functional and structural support) and four aspects (emotional, instrumental, informational, and appraisal support) [3, 4]. Social support provided by personal social networks is a prominent area of study and is becoming more widely recognized as a valuable resource for improving health, especially among individuals experiencing negative emotions [5]. Prior studies [6–12] have indicated that social support directly impacts psychological distress, depressive symptoms, life satisfaction, hope, self-transcendence, mortality risk, and so on. Furthermore, social support has been found to mediate relationships between loneliness and depression [13], mindfulness and mood [14], stress exposure and psychological distress [15], chronic diseases and positive mental health [16], competencies and job satisfaction [17], and so forth. Thus, sick or not, assessing social support is a key component in many health-related questionnaire surveys. It aims to explore the relations between social support and health and improve health levels, ultimately enhancing overall health. As for a concise tool for assessing social support, the Oslo-3 Social Support Scale (OSSS-3 or OSSS) has not been officially validated in Chinese cultural backgrounds [18]. Given that, the current research intended to validate the Chinese version of the OSSS-3.

According to social relationships and social provisions theory, social relationships typically provide social provisions [19]. Inadequacies in specific provisions may lead to multiple health issues. This could be attributed to health-related quality of life being directly influenced by social support, including aspects such as emotional support, network support, and others. Individuals with strong social support are more likely to rebuild their lives after an unfortunate incident and can find an outlet to express concerns, which is associated with a reduction in both psychological and physical disorders [19, 20]. In accordance with the model of resilience, social support influences the adjustment to stress through affecting resilience [21]. Moreover, social support not only buffers or prevents the adverse effects of stress and negative events on both psychological and physical health [19], but also helps individuals maximize the benefits of positive events, such as enhancing creativity and promoting engagement in physical activity [22–24]. Biologically speaking, Uchino et al’ study [25] has demonstrated that social support is negatively associated with inflammation, which indicates that inflammation is a key biological mechanism relating social support to disease.

Nursing students encounter many pressures, such as academic pressure, interpersonal pressure, employment pressure, economic pressure, and so forth, which easily contribute to health concerns. Nursing students have also exhibited varying degrees of mental health concerns due to experiencing quarantine and online education during the pandemic [26]. In the context of new cases and variants ebbing and flowing and a health-centered “big medicine” model, there is a growing demand for more nurses who are expected to maintain optimal health. Nevertheless, the significant prevalence of health concerns among nursing students can worsen the healthcare workforce crisis by widening the disparity between the growing need for top-notch healthcare services and the availability of competent nurses. This exacerbates the shortage in the healthcare workforce.

Rightfully so, the health status of nursing students deserves more attention. As previously mentioned, Social support has been proven beneficial for health. Assessing social support is a key component in most questionnaire surveys related to health status, aiming to enhance overall health. Regarding tools for assessing social support, commonly used instruments include the Multidimensional Scale of Perceived Social Support [27], the Duke Social Support Index [28], Sarason’s Social Support Questionnaire [29], the OSSS-3 [30, 31], and so forth. The OSSS-3, with only three items, is the most concise among these instruments. This brevity can significantly alleviate respondent fatigue from a lengthy questionnaire and enhance the quality of the study. Despite its limited item count, the OSSS-3 possesses the main aspects of social support and demonstrates favorable psychometric properties [18]. It has been widely employed in several pieces of research in different settings [18, 31–36]. Therefore, as a reliable and abbreviated instrument, the OSSS-3 is considered a choice in this study.

However, to date, the OSSS-3 has not been officially validated in Chinese cultural backgrounds. Moreover, Classical Test Theory (CTT) was commonly used to validate it in prior studies. Item Response Theory (IRT) models have not been applied for its validation. CTT is unable to evaluate the status of social support within the context of the overall degree of social support [37]. Significantly, this limitation can be addressed through the use of IRT models [38]. Item responses are used by IRT models to generate a linear scale that shows the “less” to “more” of a trait or latent variable [38]. Consequently, it may directly compare the relationship between the respondent’s position and the item’s position on the scale of that latent variable.

Given that, the current research intended to validate the Chinese version of the OSSS-3 among nursing students. Both CTT and IRT models were applied in assessing reliability, validity, discrimination, and difficulty.

## Methods

### Translation procedure and cross-cultural adaptation

**Fig. 1:**
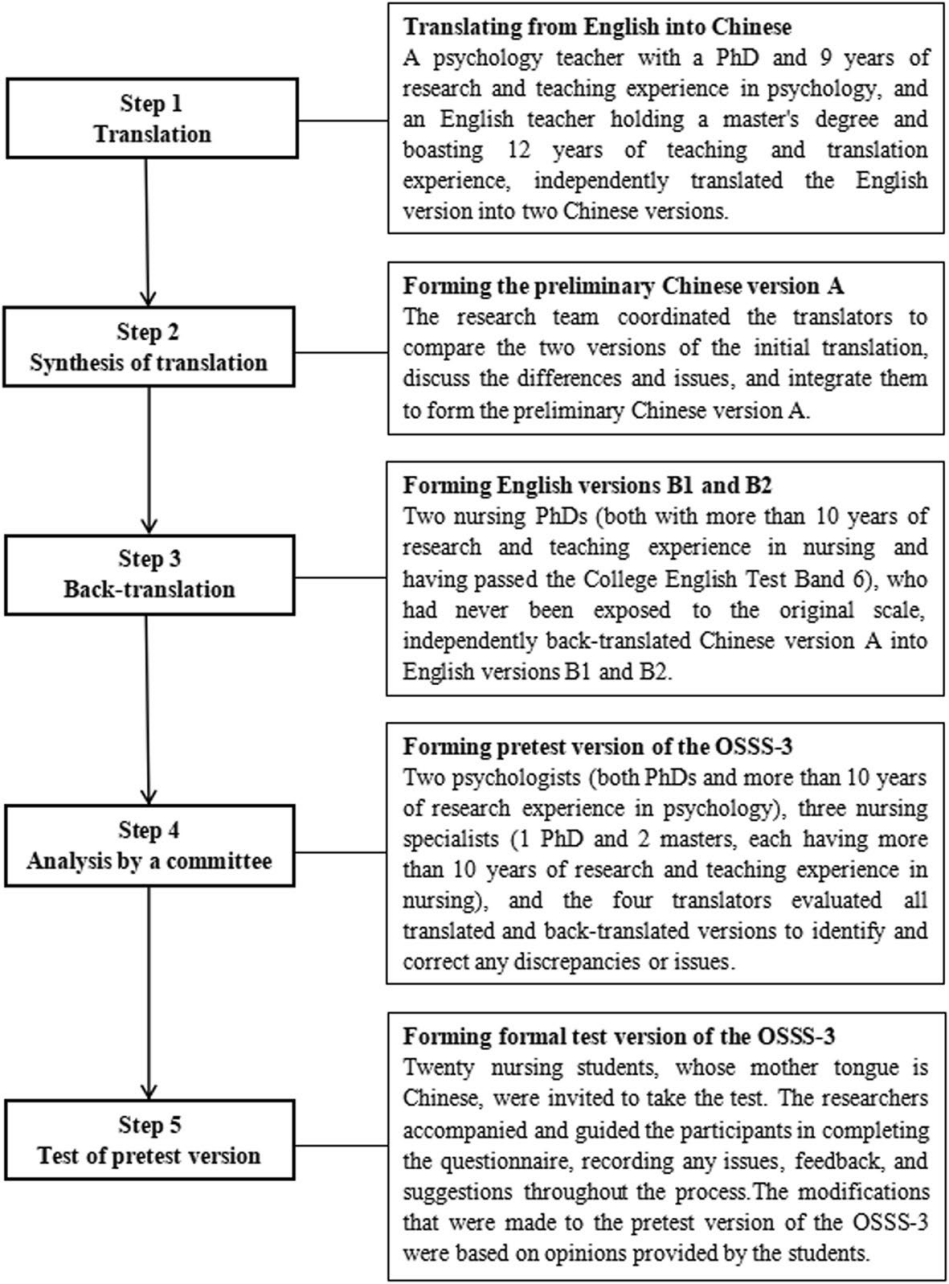
Translation and cross-cultural adaptation process

The following were the measures that we took in accordance with the translation criteria (Fig. 1) [39, 40]. Step 1, the OSSS-3 was translated from English into Chinese. A psychology teacher with a PhD and 9 years of research and teaching experience in psychology, and an English teacher holding a master’s degree and boasting 12 years of teaching and translation experience, independently translated the English version into two Chinese versions. Step 2, the preliminary Chinese version A was formed. The research team coordinated the translators to compare the two versions of the initial translation, discuss the differences and issues, and integrate them to form the preliminary Chinese version A. Step 3, the English versions B1 and B2 were formed. Two nursing PhDs (both with more than 10 years of research and teaching experience in nursing and having passed the College English Test Band 6), who had never been in contact with the scale, independently back-translated Chinese version A into English versions B1 and B2. Step 4, the pretest version of the OSSS-3 was formed. Two psychologists (both PhDs and more than 10 years of research experience in psychology), three nursing specialists (1 PhD and 2 masters, each with over 10 years of expertise in nursing research and teaching), and the four translators evaluated all translated and back-translated versions to identify and correct any discrepancies or issues. Step 5, a formal test version of the OSSS-3 was formed. Twenty nursing students, whose mother tongue is Chinese, were invited to take the test. The researchers accompanied and guided the participants in completing the questionnaire, recording any issues, feedback, and suggestions throughout the process. The modifications that were made to the pretest version of the OSSS-3 were based on opinions provided by the students.

### Participants and procedures

Following Kendall’s criterion [41], the number of samples exceeding 33 was calculated by multiplying 10–20 with the total items and adding higher than 10%, considering that the OSSS-3 comprises 3 items. The OSSS-3 was validated on nursing undergraduates from a medical university in Anhui Province. Inclusion criteria comprised full-time nursing student status and provision of consent to participate. Multistage sampling was utilized. In stage 1, the sample was stratified based on grade, covering four grades from freshman (first-year students) to senior (fourth-year students). In stage 2, random selection was used to select half of each grade’s classes. This yielded a total of 42 classes across the four grades. In stage 3, all students (25–30) from each class were sampled. This yielded a total of 1008 nursing students from the 42 classes. The questionnaires were distributed using Questionnaire Star. All respondents offered informed consent prior to data collection. Subsequently, respondents proceeded to the questionnaire-filling interface to select their options. Ultimately, we received 1008 questionnaires in April 2022. There were 966 eligible questionnaires. Furthermore, 30 nursing students were randomly chosen to complete the surveys again for evaluation stability in 10-day intervals.

### Instruments

#### Sociodemographic information

In line with previous research [42–45], demographic statistics including gender, age, academic year, and stress levels related to learning, life, and job hunting were collected.

#### OSSS-3

The OSSS-3, comprising only three items (Oslo 1, Oslo 2, and Oslo 3), demonstrates good reliability and validity and encompasses several domains of social support (Table 1). The OSSS-3 has a total score range of 3 to 14, where 3–8, 9–11, and 12–14 indicate low, moderate, and high levels of social support respectively [18]. The German version’s Cronbach’s coefficient was 0.640 [18].

**Table 1:** The OSSS-3 (English version and Chinese version)

**Item**	**Content**	**Score**
Oslo 1	How many people are so close to you that you can count on them if you have great personal problems?	1 (none), 2 (1–2), 3 (3–5), 4 (5+)
	如果你遇到了大的困难, 在你亲近的人中有多少个可以给你提供帮助?	1 (0), 2 (1–2), 3 (3–5), 4 (≥ 6)
Oslo 2	How much interest and concern do people show in what you do?	5 (a lot), 4 (some), 3 (uncertain), 2 (little), 1 (none)
	你熟悉的人对你所做的事情表现出多少关注和兴趣?	5 (很多), 4 (一些), 3 (不确定), 2 (少), 1 (没有)
Oslo 3	How easy is it to get practical help from neighbors if you should need it?	5 (very easy), 4 (easy), 3 (possible), 2 (difficult), 1 (very difficult)
	如果你需要周围熟悉人的帮助时, 你容易得到他们的帮助吗?	5 (非常容易), 4 (容易), 3 (一般), 2 (困难), 1 (非常困难)

### Statistical analysis

To analyze the statistics in the current study, SPSS 25.0, AMOS 23.0, and R 4.3.0 were applied. The reliability was evaluated by Cronbach’s α coefficient based on standardized items, split-half reliability, retest reliability, and item-total score correlations. Due to the dissimilarity in response scales between Oslo 1 (4-point Likert-type item) and the other two items (5-point Likert-type), the reliability was tested employing Cronbach’s α coefficient based on standardized items [46].

The content validity was calculated employing the content validity index (CVI). Construct validity was tested through exploratory factor analysis (EFA). Multiple-group confirmatory factor analysis (MGCFA) was adopted to test its measurement invariance across genders. Gender is a significant factor in social support, with prior studies [47, 48] suggesting that females perceive greater social support than males. Moreover, prior studies [18, 49] did not investigate potential gender invariance. Therefore, gender was chosen to test whether it had multi-group invariance in this study [50]. Model fit of MGCFA was tested employing chi-square/degree of freedom (*χ*^*2*^/*df*), goodness of fit index (GFI), adjusted goodness of fit index (AGFI), root-mean-square error of approximation (RMSEA), incremental fit index (IFI), Tucker-Lewis index (TLI), and comparative fit index (CFI). GFI, AGFI, IFI, TLI, and CFI have recommendations that are greater than 0.90 [51]. The criteria for *χ*^2^/*df* falls below 5 [51]. The criteria for RMSEA falls below 0.08 [51]. The change in Chi-square values (Δ*χ*^2^) serves as the primary indicator of invariance. The non-significant values of Δ*χ*^2^ (*P* > 0.05) indicate group invariance [1, 25, 51]. The difference in latent means of social support construct between different groups was assessed by the Critical Ratio (CR), with an absolute value being less than 1.96 indicating equality between the parameters. Discriminant validity was evaluated employing an independent samples t-test.

The Graduated Response Model (GRM) was employed according to IRT models. The discrimination parameters (α) and difficulty parameters (β) were calculated for every single item. The α value falls within the range of 1.35 to 1.69, indicating extremely high, and above 1.70, indicating very high [52]. According to the difficulty criterion, values should exhibit a monotonic increase [53, 54]. Measurements included item characteristic curves (ICC) and total information curves (TIC).

### Ethical considerations

This research was authorized by the ethical committee of the College of Nursing at Wannan Medical College (20,220,004). All procedures were conducted based on relevant guidelines and regulations. Prior to data collection, all enrolled nursing students provided their informed consent. Within the informed consent process, all participants were briefed on the research objectives and assured of their anonymity.

## Results

### Translation and cross-cultural adaptation results

Following expert feedback obtained during the translation and cultural adaptation process, along with comprehensive feedback from research team deliberations and pretest results, several key modifications were implemented: (1) Certain terms were adjusted to better align with Chinese language conventions. For instance, in the fourth response of Oslo 1, “5+” was revised to “≥6”; in the fourth response of Oslo 3, “possible” was changed to “general”. (2) Certain expressions were modified to better suit the cultural context of China. In China, residential neighborhoods have shifted away from traditional social interaction patterns among urban dwellers, potentially leading to a sense of detachment within communities due to reforms in the housing system [55]. Therefore, the expression of “people” in Oslo 2 was replaced with “familiar people " and the expression of “neighbors” in Oslo 3 was replaced with “familiar people in the vicinity”.

### The sample

The ages of 966 nursing students were between 16 and 25, with an average age of 20.42. The majority of these students were females (745, 77.12%). There were 293 freshmen (30.33%), 207 sophomores (21.43%), 277 juniors (28.67%), and 189 seniors (19.57%). The numbers with low, moderate, and high academic pressure were 150 (15.53), 439 (45.44), and 377 (39.03), respectively. The numbers with low, moderate, and high job-hunting stress were 154 (15.94), 348 (36.03), and 464 (48.03), respectively. The numbers with low, moderate, and high life stress were 160 (16.56), 471 (48.76), and 335 (34.68), respectively. Moreover, those categorized as low, moderate, and high social support of nursing students were 329 (34.06%), 513 (53.11%), and 124 (12.84%), respectively.

Among the 30 nursing students chosen for retesting, their ages were between 18 and 24, with an average age of 20.60. The majority of them were females (23, 76.67%). There were 8 freshmen, 8 sophomores, 7 juniors, and 7 seniors. Those categorized as low, moderate, and high social support of nursing students were 9 (30.00%), 18 (60.00.11%), and 3 (10.00%), respectively.

### Reliability

Among nursing students, the split-half reliability was 0.622. The Cronbach’s α coefficient based on standardized items of the OSSS-3 was 0.687. When the item was removed, the Cronbach’s α coefficients were between 0.527 and 0.640, all lower than the overall Cronbach’s α coefficient. The corrected item-total correlations varied between 0.449 and 0.543. Moreover, the correlations between individual items and total scores varied from 0.723 to 0.835 (Table 2). The retest coefficient was 0.907.

**Table 2:** Item characteristics of the OSSS-3

**Item**	***M (SD)***	**Cronbach’s α coefficient if the item deleted**	**Corrected item-total correlation**	**Item-total correlation**
Oslo 1	2.70 (0.77)	0.640	0.449	0.723^***^
Oslo 2	3.12 (1.03)	0.579	0.514	0.835^***^
Oslo 3	3.45 (0.80)	0.527	0.543	0.789^***^
OSSS-3	9.27 (2.05)			

***P < 0.001

### Validity

#### Content validity

The item CVI (I-CVI) of Oslo 1, Oslo 2, and Oslo 3 were 1.000, 0.800, and 1.000 respectively. The average I-CVI for all individual items (S-CVI_Ave_) was 0.933.

### Construct validity

#### Exploratory factor analysis

In the EFA, the Kaiser–Meyer–Olkin value (KMO) was 0.658 and the Bartlett spherical test value was 492.015 (*df* = 3, *P* < 0.001). These results suggested that factor analysis can be performed [46]. The results revealed the extraction of a single common factor, explaining 61.559% of the entire variance. The loading values for Oslo 1, Oslo 2, and Oslo 3 were between 0.743 and 0.814. The communalities for Oslo 1, Oslo 2, and Oslo 3 were between 0.552 and 0.663 (Table 3).

**Table 3:** Factor load and communality

	**Factor load values**	**Communalities**
Oslo 3	0.814	0.663
Oslo 2	0.795	0.632
Oslo 1	0.743	0.552
Eigenvalue	1.847	
% of variance	61.559	

#### Multiple-group confirmatory factor analysis

**Fig. 2:**
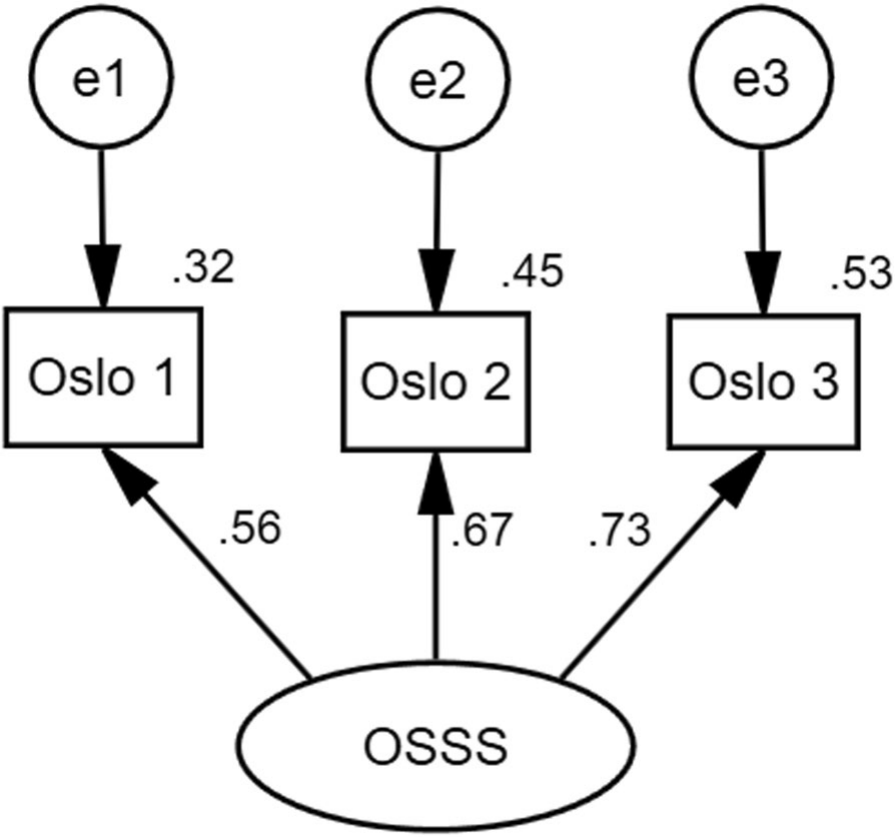
The multiple-group confirmatory factor analysis model by gender

In the MGCFA, the model of measurement weights displayed a good goodness-of-fit, with *χ*^2^ = 1.711, *df* = 2, *P =* 0.425, *χ*^2^/*df* = 0.855, GFI = 0.999, AGFI = 0.993, RMSEA = 0.029, IFI = 1.000, TLI = 1.000, and CFI = 1.000 (Fig. 2). Δ*χ*^2^ had no significant change (*P* = 0.055), which indicated gender invariance. Also, the absolute values of CRs were all below 0.001 and were deemed equivalent between the parameters.

#### Discriminant validity

The difference in scores between the top (30%) and bottom (30%) groups attained significance (Table 4).

**Table 4:** Discriminant validity analysis

**Item**	**Low-score group**	**High-score group**	***t***	***P***
Oslo 1	2.09 ± 0.53	3.18 ± 0.64	26.076	< 0.001
Oslo 2	2.12 ± 0.66	3.84 ± 0.73	34.677	< 0.001
Oslo 3	2.79 ± 0.71	3.98 ± 0.55	25.318	< 0.001

#### Item response theory models

**Fig. 3:**
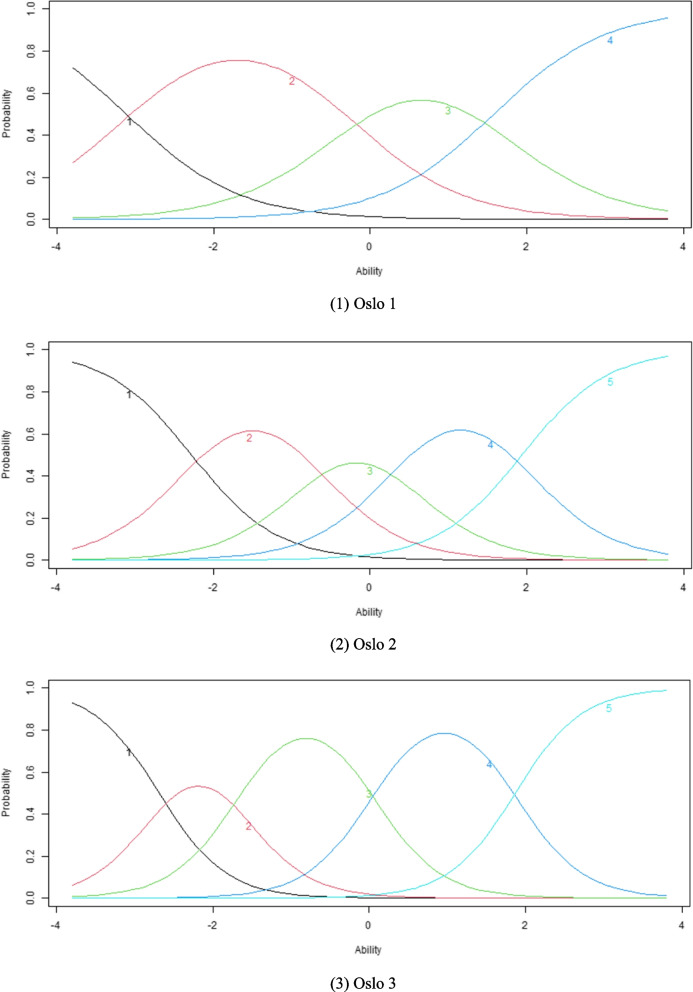
Item characteristic curves. Oslo 1, Oslo 2, Oslo 3

**Fig. 4:**
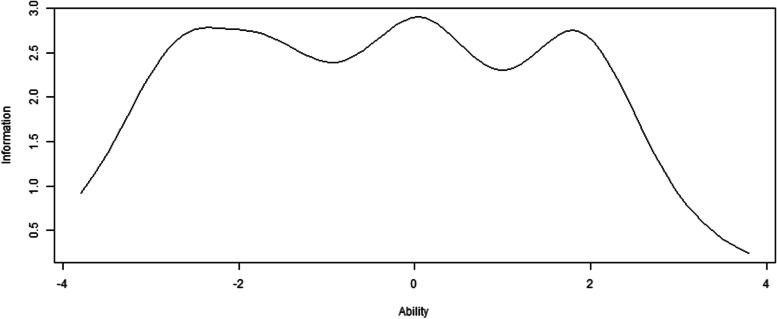
Total (scale) information curves

 The EFA results confirmed the single dimension and local independence, supporting the applicability of the GRM. The α values of the OSSS-3 varied from 1.39 to 2.33 (Table 5). The β values of the OSSS-3 exhibited a monotonically increasing pattern from β1 to β3 (Oslo 1) and from β1 to β4 (Oslo 2 and Oslo 3). The ICC presented the likelihood of an individual selecting a particular category at a given latent construct level. Every curve represents a possible reaction (Fig. 3). As indicated by the TIC, the test exhibited higher precision within the range of -3 to 2.5, reaching its peak at an ability value of 0 (Fig. 4).

**Table 5:** Discrimination and difficulty parameters

**Item**	**α**	**β**_**1**_	**β**_**2**_	**β**_**3**_	**β**_**4**_
Oslo 1	1.39	-3.01	-0.27	1.58	
Oslo 2	1.84	-2.28	-0.72	0.37	1.94
Oslo 3	2.33	-2.69	-1.67	0.05	1.87

## Discussion

Up to this point, no studies have been conducted to validate the OSSS-3 using IRT models, and an officially validated Chinese version has not been identified. Therefore, this research intended to verify the Chinese version of the OSSS-3 among nursing students. Both CTT and IRT models were employed to test its reliability, validity, discrimination, and difficulty. The findings confirmed that the Chinese version of the OSSS-3, a concise and cost-effective self-assessment instrument, possesses satisfactory psychometric properties, making it suitable for assessing nursing students in China.

As for reliability, Cronbach’s α coefficient falls within the range of 0.600-0.699 and 0.700-0.799 are considered acceptable and good, respectively [46]. This research yielded acceptable results. In our research, the Cronbach’s α coefficient (0.687) was significantly higher than the Nigeria version (0.500) and slightly higher than the German version (0.640) [18, 49]. Generally speaking, the Cronbach’s α coefficient decreases when the number of items is reduced [56]. Moreover, a relatively lower value does not necessarily imply low reliability but rather suggests multidimensionality [49]. Despite consisting of only three items, its Cronbach’s α coefficient in our research approached 0.700. Therefore, the Chinese version of OSSS-3 can be regarded as possessing good internal consistency. Furthermore, all corrected item-total score correlations exceeded 0.4, which validated that the psychological or underlying traits to be measured are highly homogenous between items [46]. Every item’s correlation with the overall scores was significantly higher than 0.4, which indicated there is excellent homogeneity between all items and the scale. Moreover, the retest coefficient of the OSSS-3 confirmed excellent stability.

In assessing content validity, CVI is rated as good when both I-CVI and S-CVI_Ave_ are not less than 0.78 and 0.90, respectively [57]. In the current study, both the I-CVI and S-CVI_Ave_ exceeded these thresholds, indicating satisfying content validity. This signifies the appropriateness and agreement of the content being measured.

In the EFA, it is recommended that a single item’s loading value should be at least 0.40 on the common factor [58]. Meanwhile, Additionally, the combined contribution of the extracted common factors should exceed 40% [58]. The Chinese version of OSSS-3 was a single-dimensional scale, which was in accordance with the other versions [18, 49]. The single factor explained 61.559% of the entire variance, surpassing the German version (58.54%). Each item in the OSSS-3 exhibited an ideal load value. The communality coefficients all exceeded 0.4. All these validated the Chinese version of OSSS-3 possesses satisfying construct validity [18, 46]. In the MGCFA, the analysis of invariance indicated that the evaluation of social support by the OSSS-3 was consistent across genders, with each item representing the same construct for both males and females [51].

In the IRT models, Oslo 1 demonstrated high discrimination, while Oslo 2 and Oslo 3 exhibited very high discrimination because of the α values exceeding 1.35 and 1.70, respectively. These findings indicated that the OSSS-3 effectively discriminates levels of social support [59]. More specifically, the OSSS-3 can readily differentiate between individuals possessing high, moderate, and low social support. Regarding difficulty, the β values validated that the OSSS-3 possesses an appropriate degree of difficulty [19]. To be specific, individuals who have low social support will be more likely to choose the lower response, while those with higher social support will be more likely to choose the higher response [59]. In terms of ICC, the findings revealed the response choices for each of the corresponding items being monotonically correlated with social support. Social support grows as one moves from left to right on the x-axis.

The OSSS-3 is a fairly practical instrument. Notably, it stands out for its simplicity. Similar to prior studies [18, 31, 49], it requires less than a minute to complete, minimizing respondent burden and enhancing participant compliance. Moreover, it is a reliable tool. This current study not only validated the satisfactory reliability and validity of OSSS-3 but also satisfactory discrimination, difficulty, and accuracy. Consequently, the Chinese version of OSSS-3 emerges as a suitable instrument for assessing social support among nursing students. In nursing education, research, and practice, the utilization of the OSSS-3 contributes to enhancing students’ academic performance and psychological well-being. For instance, by employing the OSSS-3, educators can explore the relations and mechanisms between support received from peers and teachers and the teaching effectiveness in blended learning. This can serve as a foundation for educational reforms. Researchers can explore the relations and mechanisms between social support and psychological well-being, thereby establishing a scientific foundation for enhancing nursing students’ psychological health [12, 60]. Moreover, the health authorities can examine the relations and mechanisms between social support and vocational emotion in the work environment of nursing interns. This will facilitate timely implementation of strategies and coping mechanisms to enhance nursing students’ professional identity and cultivate high-quality nursing reserves.

### Limitations and strengths

The participants were nursing students from the same institution. As a result, future research should broaden the sample’s range. The Chinese version of the OSSS-3 was tested among nursing students. This may restrict the use of OSSS-3 in other populations. Therefore, it is imperative for future studies to examine the instrument’s applicability across diverse demographic groups. These efforts will provide additional evidence for the application of the OSSS-3 and establish a robust scientific foundation for measuring social support. Moreover, the utilization of self-report measures may lead to response biases. Albeit these disadvantages, this study stands out as pioneering in comparison with earlier research. Notably, it marks the first instance of applying IRT models to validate the OSSS-3. Additionally, the research validates a concise and practical tool to assess social support. This instrument can be integrated into future research without imposing a substantial burden on both participants and researchers, ultimately enhancing the quality of the study.

## Conclusion

Nursing students encounter various pressures that can contribute to health issues. Social support is beneficial for promoting well-being. Assessing social support is essential for enhancing health outcomes. The OSSS-3, a concise and widely used tool for measuring social support, was utilized in this study to evaluate its psychometric properties among Chinese nursing students. The results verify the OSSS-3 possesses good homogeneity, stability, validity, discrimination, and difficulty. Moreover, this instrument is practical in assessing social support, requiring minimal additional effort from participants and researchers. Given that, the Chinese version of OSSS-3 is a proper instrument for testing nursing students’ social support. Future research should expand the study range and include a more diverse population to further validate the OSSS-3. These efforts will provide additional evidence for the application of the OSSS-3 and establish a robust scientific foundation for measuring social support.

## Data Availability

The datasets generated and/or analyzed during the current study are available from the corresponding authors.
